# How much is a pheromone worth?

**DOI:** 10.12688/f1000research.9195.1

**Published:** 2016-07-20

**Authors:** Jose Mauricio S. Bento, Jose Roberto P. Parra, Silvia H. G. de Miranda, Andrea C. O. Adami, Evaldo F. Vilela, Walter S. Leal

**Affiliations:** 1 Department of Entomology and Acarology, University of São Paulo, ESALQ, Piracicaba, Brazil; 2 Department of Economics, Administration and Sociology, University of São Paulo, ESALQ, Piracicaba, Brazil; 3 Department of Entomology, Federal University of Viçosa, Viçosa, Brazil; 4 Department of Molecular and Cellular Biology, University of California, Davis, CA, USA

**Keywords:** Gymnandrosoma aurantianum, citrus fruit borer, benefit-cost analysis, pest management, monitoring

## Abstract

Pheromone-baited traps have been widely used in integrated pest management programs, but their economic value for growers has never been reported.  We analyzed the economic benefits of long-term use of traps baited with the citrus fruit borer *Gymnandrosoma aurantianum *sex pheromone in Central-Southern Brazil. Our analysis show that from 2001 to 2013 citrus growers avoided accumulated pest losses of 132.7 million to 1.32 billion USD in gross revenues, considering potential crop losses in the range of 5 to 50%. The area analyzed, 56,600 to 79,100 hectares of citrus (20.4 to 29.4 million trees), corresponds to 9.7 to 13.5% of the total area planted with citrus in the state of São Paulo. The data show a benefit-to-cost ratio of US$ 2,655 to US$ 26,548 per dollar spent on research with estimated yield loss prevented in the range of 5-50%, respectively. This study demonstrates that, in addition to the priceless benefits for the environment, sex pheromones are invaluable tools for growers as their use for monitoring populations allows rational and reduced use of insecticides, a win-win situation.

## Introduction

The discovery of bombykol as the sex pheromone of a domesticated insect species ( Butenandt *et al.*, 1959) triggered the interest of entomologists and natural product chemists to jointly identify pheromones from economically important insect pests and explore their potential in Integrated Pest Management (IPM) ( Howse *et al.*, 1998; Ridgway *et al.*, 1990; Silverstein, 1981). This interest continues to increase to date given the need for environmentally friendly alternatives to control insect pest populations. After all, pheromones are non-polluting and usually non-toxic natural products. Strictly speaking they are nature-inspired synthetic compounds, i.e., identical to natural products, but manmade chemical signals. Additionally, pheromones are species-specific and safe for beneficial organisms; thus, they are ideal components of IPM programs ( Jutsum & Gordan, 1989). Of note, pheromones have been registered in many countries for use in pest management, and no evidence of adverse effects has been reported ( Witzgall *et al.*, 2010). There is a consensus that successful implementation of pheromones in the field frequently involves a joint effort by chemical ecologists, entomologists and/or extension agronomists, and growers, in addition to the pheromone industry ( Witzgall *et al.*, 2010).

There are many ways in which pheromones can be used for surveillance and IPM programs, including monitoring, attract-and-kill, and mating disruption. Pheromone-baited traps are sufficiently sensitive to detect low population densities and are therefore an effective way for tracking invasive species while they are still at the establishment stage ( El-Sayed *et al.*, 2006; Liebhold & Tobin, 2008). Population monitoring has been a simple and widely used strategy to determine the ideal moment for application of control procedures (i.e., insecticides), using pre-defined thresholds (action levels) based on level of capture in pheromone-baited traps. This strategy reduces the use of insecticides to the minimal amount necessary to protect both crops and the environment ( Thomson *et al.*, 1999; Witzgall *et al.*, 2010).

One of the first systems to use an action level based on capture with pheromone-baited traps was established for the pea moth *Cydia nigricana* ( Wall *et al.*, 1987). Later, many other studies were conducted with equal success in agricultural, horticultural or forestry applications, against pest species including the European corn borer *Ostrinia nubilalis* ( Laurent & Frérot, 2007), tufted apple budmoth *Platynota idaeusalis* ( Knight & Hull, 1989), lightbrown apple moth ( Bradley *et al.*, 1998), scale insects ( Dunkleblum, 1999), Mullein bug *Campylomma verbasci* ( McBrien *et al.*, 1994), grapevine moth *Lobesia botrana* ( Ioriatti *et al.*, 2011), codling moth *Cydia pomonella* ( Madsen & Vakenti, 1973), Oriental fruit moth *Grapholita molesta* ( Rothschild & Vickers, 1991), pink bollworm *Pectinophora gossypiella* ( Qureshi *et al.*, 1984), Old World bollworm *Helicoverpa armigera* ( Cameron *et al.*, 2001), cotton leafworm *Spodoptera litura* ( Singh & Sachan, 1993), and yellow rice stem borer *Scirpophaga incertulas* ( Krishnaiah *et al.*, 1998), just to cite a few.

Despite the clear advantages offered by the use of pheromones in IPM in recent decades, particularly the use of traps for monitoring in extensive areas, to date there are no studies on their economic benefits. While the benefits for the environment are less tangible, the economic benefits could be estimated. Evidence of economic benefits could be extremely helpful in motivating growers to employ environmentally friendly strategies for pest control, the chemical industry to participate in production and commercialization of pheromones, and the public and private sector to promote and support more translational research.

The citrus fruit borer *Gymnandrosoma aurantianum* Lima (Lepidoptera, Tortricidae) is a representative case for analysis of the economic benefits achieved by the use of a synthetic pheromone to manage this pest in extensive areas. Brazil is the leading worldwide producer of citrus ( USDA, 2015), and Central-Southern Brazil, an area with generalized occurrence of the citrus fruit borer, accounts for approximately 80% of all citrus production in the country ( IBGE, 2015). Females normally deposit a single egg per fruit ( Garcia & Parra, 1999); after eclosion, the larvae pierce the skin and bore into the fruit in order to feed on the pulp ( Fonseca, 1934). Once they have penetrated the fruit, larval control becomes impracticable and the fruit is rendered unfit for consumption ( Bento *et al.*, 2001).

In the 1980s, indiscriminate use of insecticides, especially pyrethroids, against a wide variety of pest insects and mites in citrus orchards in Central-Southern Brazil contributed to a drastic reduction of natural enemies, favoring an increase in the population of *G. aurantianum* ( Parra *et al.*, 2004). Starting in the 1990s, yield losses due to the citrus fruit borer were estimated at over US$50 million per year ( Anonymous, 2000).

The sex pheromone of *G. aurantianum*, ( *E*)-8-dodecenyl acetate and ( *E*)-8-dodecenol, was identified by members of our group in early 2000’s ( Leal *et al.*, 2001). At that time, Bento *et al.* (2001) established strategies for its use in the field, including the number of traps per area, trap positioning on trees, pheromone durability, and control level based on number of males collected per week. In November 2001, the Rural Growers Cooperative (Coopercitrus) placed the synthetic pheromone on the market, focusing on citrus growers in the state of São Paulo after an intense campaign to divulge the technology, train extension agronomists, give presentations, and distribute technical bulletins to citrus producers ( Parra *et al.*, 2004).

In this paper, we report a benefit-cost analysis applied to the citrus industry in the period from 2001 to 2013 in the state of São Paulo, Brazil, based on gross revenues (in US$) corresponding to total production (in boxes of oranges) that growers avoided losing by using traps baited with the sex pheromone of the citrus fruit borer *G. aurantianum* in the monitored areas. We also discuss strategies for pheromone-baited trap use and its efficiency in the management and control of *G. aurantianum*.

## Materials and methods

### Benefit-cost analysis

The economic analysis covered the period from November 2001 to December 2013. Monetary results were calculated as losses avoided, i.e., the amount of gross revenues (in US$) corresponding to total production (in boxes of oranges) whose loss was prevented by using traps baited with pheromone of the citrus fruit borer *G. aurantianum*, in the monitored areas in the state of São Paulo, Brazil.

Data on the number of citrus trees in the state of São Paulo and their average yield (boxes/tree) were obtained from the Agricultural Economics Institute (IEA) ( IEA, 2015). To calculate the average annual price (US$) of sale of one box of oranges (40.8 kg), we used the average monthly price published by the Center for Advanced Studies on Applied Economics (Cepea) ( Cepea, 2015), corresponding to the average amounts in US$ paid to citrus growers per box, on credit, in the state of São Paulo, Brazil, including costs of harvesting and shipping, for oranges of the Pera, Natal and Valencia varieties. Monetary variables were updated to values applicable in June 2014, the final month of data used in this report, using the average exchange rate (PTAX) effective on that month as informed by the Central Bank of Brazil ( Bacen, 2015). The reference discount rate considered here was the average annual rate of 4% published by the Special System for Settlement and Custody (Selic) of the Central Bank of Brazil for June 2014 ( Bacen, 2015), and the nominal data were transformed into real values using the General Price Index – Internal Availability (IGP-DI), published by the Getúlio Vargas Foundation ( FGV, 2015).

The number of traps baited with *G. aurantianum* pheromone sold between November 2001 and December 2013, as well as their prices (in US$) were obtained from the Coopercitrus, the only entity responsible for their distribution in the entire state of São Paulo, Brazil. Each year (2001–2013), *G. aurantianum* was monitored during the citrus harvesting season (~ 6 months). According to Bento *et al.* (2001), the traps have a durability of one month and cover an area of approximately 10 hectares when used for monitoring. Therefore, six traps/year were installed per 10 hectares monitored. According to available data, the citrus fruit borer can cause yield losses of up to 50% per tree ( Parra *et al.*, 2004). However, for our calculations, we considered a 5 to 50% range of losses avoided in the period from November 2001 to December 2013. Costs were calculated based on the prices paid for purchase of the traps and the initial amount invested in research to develop the technology, which was US$50,000 ( Parra *et al.*, 2004). Costs of labor for trap installation and monitoring, as well as indirect investments, including use of University resources and researchers and product registration expenses, were not taken into account. Benefits were estimated in the form of losses avoided, by calculating the number of boxes produced in a scenario in which the citrus fruit borer is present, i.e., considering the yield losses that would be caused by the pest if no traps had been used. These losses were then monetized, based on the price of a box of oranges. Finally, the benefit-to-cost ratio was calculated based on total present value, considering both the benefits and the estimated costs of monitoring and control of the citrus fruit borer between 2001 and 2013.

## Results and discussion

**Figure 1.**
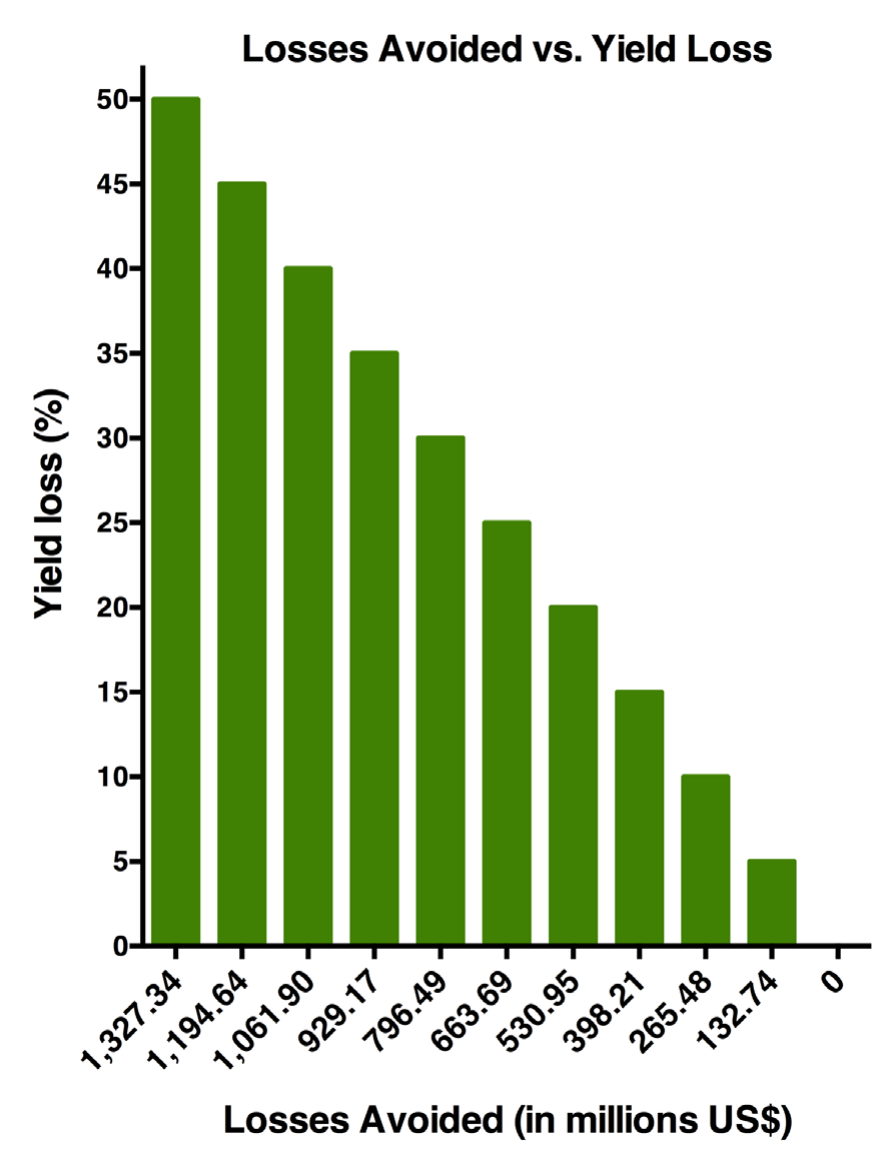
Losses avoided (in millions US$) by using traps baited with the sex pheromone for the citrus fruit borer *Gymnandrosoma aurantianum* between 2001 and 2013 in the state of São Paulo, Brazil. Calculations considered yield loss ranging from a very conservative (5%) up to high (50%) estimates ( Parra *et al.*, 2004).

Total losses avoided by using traps baited with sex pheromone of *G. aurantianum* in the period from 2001 to 2013 ranged from US$132.7 million to US$1.32 billion in gross revenues. In other words, this was the estimated aggregate total of gross revenues from the sale of oranges that growers avoided losing by using pheromone-baited traps, considering a 5–50% range of potential losses caused by citrus fruit borer infestation in citrus orchards in the state of São Paulo ( Table 1; Figure 1). Of note, it is already known that the citrus fruit borer can cause yield losses of up to 50% per tree ( Parra *et al.*, 2004)

**Table 1.** Data on use of traps baited with synthetic pheromone of the citrus fruit borer *Gymnandrosoma aurantianum*, in relation to the total area planted with citrus in the state of São Paulo, Brazil, between November 2001 and December 2013.

	2001 ^*^	2002	2003	2004	2005	2006	2007	2008	2009	2010	2011	2012	2013
***Citrus data***													
Area (ha)	581,487	586,837	600,06	587,935	574,51	571,532	584,096	592,566	551,901	548,103	563,952	470,082	455,000
Trees (units)	205,811,063	211,631,592	212,560,034	215,424,155	215,030,451	211,084,838	217,485,693	231,763,878	225,665,723	211,425,179	224,716,022	215,616,377	194,740,000
Trees (units/ ha)	353.94	360.63	354.23	366.41	374.28	369.33	372.35	391.12	408.89	385.74	398.47	458.86	428.00
Yield (boxes/ tree)**	1.82	1.77	1.93	1.74	1.92	1.92	1.95	1.99	1.87	1.86	1.77	1.90	1.91
Yield (boxes/ ha)**	645.91	639.98	682.37	639.22	717.67	707.38	724.67	779.90	763.38	717.13	704.32	872.32	819.57
***Trap data***													
Traps sold (units)	7,824	33,996	40,828	44,752	45,576	41,052	47,436	35,874	33,570	31,970	33,616	35,398	33,924
Area covered by traps (ha)***	13,040	56,660	68,047	74,587	75,960	68,420	79,060	59,790	55,950	53,283	56,110	58,997	56,507
Trees covered by traps ****	4,615,368	20,433,350	24,104,259	27,329,160	28,430,685	25,269,669	29,437,659	23,385,011	22,877,286	20,553,506	22,357,959	27,060,486	24,184,853
Area covered by traps (%)	2.24	9.66	11.34	12.69	13.22	11.97	13.54	10.09	10.14	9.72	9.95	12.55	12.42

*Start sales (Nov., 2001) ** 1 box = 40.8 Kg *** 1 trap/10ha/month, during 6 months (see Bento *et al.*, 2001)(Trap sold per year/6 × 10ha) **** Area covered by traps (ha)/year × Trees (units/ha/year)

The total cost of trap purchases from 2001 to 2013 was US$5,065,807.81 (US$5.06 million). It should be noted that some costs were not measured in this study, such as labor costs of the inspections that used to be performed before the traps became available, and the fact that insecticide spraying was once triggered by a 3–5% yield loss caused by the caterpillars of *G. aurantianum* ( Gravena, 1998). Therefore, economic losses due to infested fruit were already occurring in the field, as were expenditures on chemical controls (labor, products and machine time) that were extensively used in the entire area of the orchard. Pheromone-baited traps lowered the costs of inspections (labor) in the entire orchard, in addition to reducing the costs of machine operation and insecticide use, as chemical control became targeted only at areas effectively infested with the insect at quantities above the control level. According to Bento *et al.* (2001), the use of pheromone-baited traps was shown to be efficient because it monitors adults at their mating stage, enabling growers to apply chemical control before oviposition and subsequent damage to fruits. The authors also showed that, when a control level of six or more males/week was adopted, the average percentage of damaged fruits was 0.6% in the monitored areas. In addition, after successive years of trap use, growers achieved a reduction of approximately 50% in insecticide use to control the citrus fruit borer *G. aurantianum* ( Parra *et al.*, 2004).

**Figure 2.**
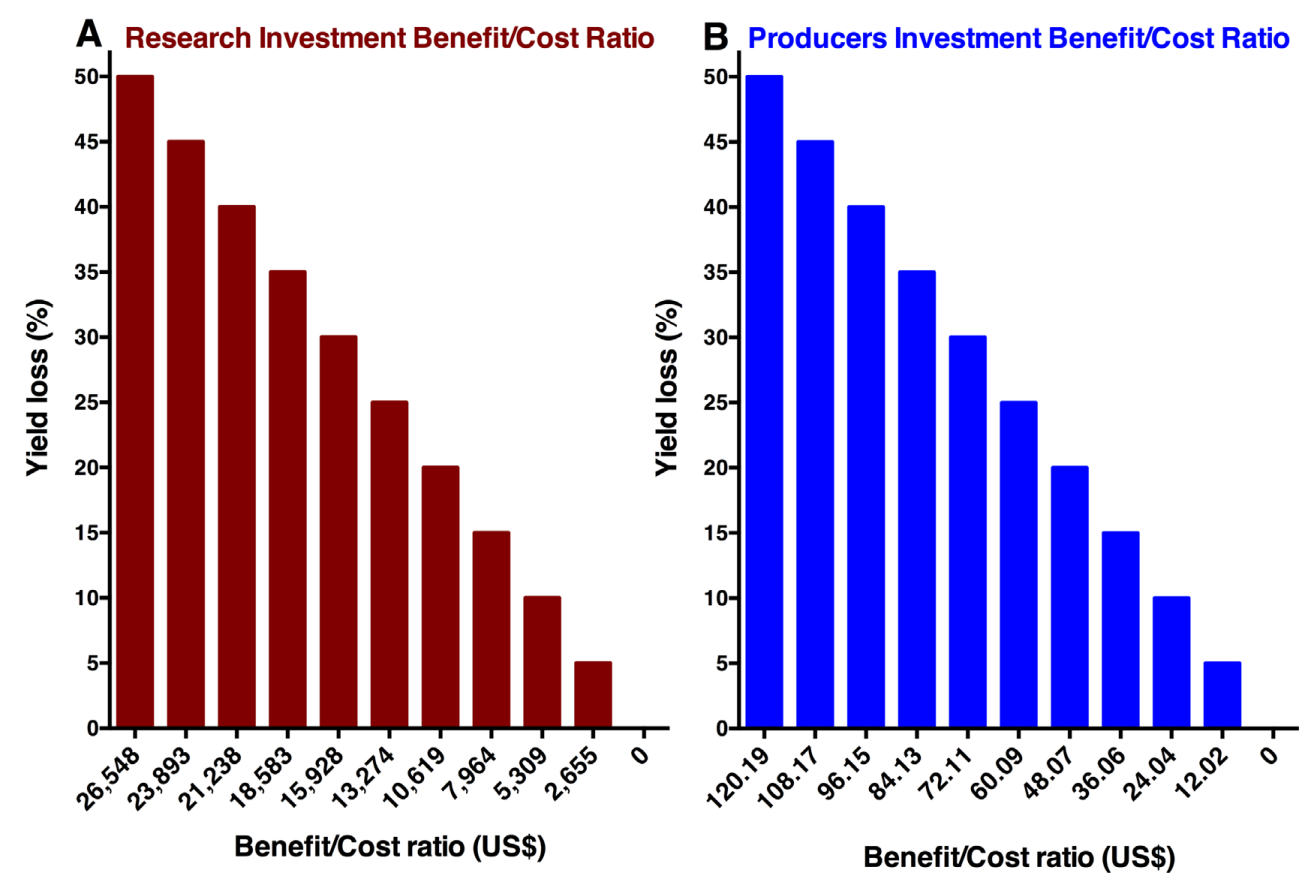
Governmental ( **A**) and producers ( **B**) benefit-to-cost ratio (US$) by investment in research or implementation of pheromone-baited traps to monitor populations of the citrus fruit borer *Gymnandrosoma aurantianum* between 2001 and 2013 in the state of São Paulo, Brazil and rationalize insecticide sprays.

The initial investment in the research that resulted in the development of pheromone-baited trap was US$ 50,000. Therefore, in terms of the governmental costs, the benefit-cost ratio of the initial investment (present value of losses avoided/total investment) ranged from US$ 2,655 to 26,548 per dollar spent with a yield loss of 5–50%, respectively ( Figure 2a). In terms of the return for the producer, in which the cost of the traps is included (US$ 5.06 million), the benefit-cost ratio was US$ 12.02 to 120.19 per dollar spent considering yield losses of 5–50% ( Figure 2b). These potential losses were based on an estimation of infestation by *G. aurantianum* in citrus orchards in the state of São Paulo.

Except for the year 2001, when the sex pheromone of *G. aurantianum* only became available on the market in November, the area monitored during the 12 subsequent years (2002–2013) ranged from 56,600 to 79,100 hectares of citrus (20.4 to 29.4 million trees), corresponding to 9.7 to 13.5% of the area planted with citrus in the state of São Paulo, the main producing region in Brazil.

These findings reveal a regularity in the sale and use of pheromone-baited traps by citrus growers during that period (2002–2013). Trap sales were relatively stable in that period, with 38,166 units sold per year on average, ranging from 31,970 units (2010) to 47,436 units (2007), possibly due to fluctuations in international prices of orange juice, the main product exported by the Brazilian citrus industry. This regularity suggests a good level of acceptance and application of the technology by growers, and certainly a benefit obtained from its use.

It worth mentioning that, according to Parra *et al.* (2004), the total volume of insecticide sprayed in the monitored areas fell by at least 50%. This can possibly be explained by the fact that spraying was only performed in areas (~10 ha) where the pest was found at levels exceeding the damage level thus preserving the other areas and, consequently, the natural enemies within them. In summary, the use of pheromone in traps for monitoring populations of the citrus fruit borer in 12 years led to tangible benefits to growers and priceless environmental savings.

Raw data for Figure 1 and Figure 2 Dataset: data used for economic analysis ( Figure 1, Figure 2A and Figure 2B) covered the period from November 2001 to December 2013 (including a combination with Table 1). Click here for additional data file. Copyright: © 2016 Bento JMS et al. 2016 Data associated with the article are available under the terms of the Creative Commons Zero "No rights reserved" data waiver (CC0 1.0 Public domain dedication).

## Data availability

The data referenced by this article are under copyright with the following copyright statement: Copyright: © 2016 Bento JMS et al.

Data associated with the article are available under the terms of the Creative Commons Zero "No rights reserved" data waiver (CC0 1.0 Public domain dedication). 

F1000Research: Dataset 1. Raw data for Figure 1 and Figure 2, 10.5256/f1000research.9195.d129239 ( Bento *et al.*, 2016).
