# Supplementary material for: How much is a pheromone worth?
Source: F1000Res. 2016 Jul 20;5:1763. [Version 1] doi: 10.12688/f1000research.9195.1 (PMC4972084; doi:10.12688/f1000research.9195.1)
Supplement: Peer review history

# Peer review: Referee response for version 1

**DOI:** 10.5256/f1000research.9897.r15333

**Published:** 2016-08-01

**Authors:** Baldwyn Torto, Jimmy Pittchar

**Affiliations:** 1 Behavioural and Chemical Ecology Department, International Centre of Insect Physiology and Ecology, Nairobi, Kenya; 2 International Centre of Insect Physiology and Ecology, Nairobi, Kenya

We considered the following issues in our review of the manuscript:

1. Economic analysis was based on direct gross revenue corresponding to total production loss avoided. How was this directly or indirectly attributed to sex pheromones?

1. Direct economic benefits are ascribed to costs avoided and cost-benefit ratio per dollar spent on research. What were direct and indirect research costs? How were these measured?

1. The accuracy of monetary values is in part, but importantly, dependent on the accurate treatment of price data. On the pivotal question of price, the reviewer considered the following questions:
  -   Is the average price decomposed into the different contributing variables?
  -   What were the relative contributions of each monetary variable to the aggregated components?
  -  What was the price elasticity?
  -   to what extent did the assigned exchange rate affect the overall pricing?
  -   how stable was the exchange rate over the entire reference period?
2. Additional benefits: environmental and ecosystem services?

**Reviewers’ specific Comments**:

1. The paper is well-written and significant because of the global renewed focus on alternative environmentally-sustainable IPM strategies using non-polluting, non toxic nature-inspired synthetic products, reducing the use of insecticides (and thereby the carbon footprint of their production) in crop and environmental protection.
2. The research methodology is adequate. The sample size is adequately representative (9.7% - 13.5% of the total area planted with citrus in Sao Paulo).
3. The use of an action level data based on capture with pheromone-baited traps is sufficiently documented/ well exemplified.
4. The main thrust of the paper **data analysis** is based on benefit-cost analysis applied to the citrus industry in the period from 2001 to 2013 in the state of São Paulo, based on gross revenues (in US$) corresponding to total production (in boxes of oranges) that growers avoided losing by using traps baited with the sex pheromone of the citrus fruit borer *G. aurantianum *in the monitored areas. Whereas the economic value of the pheromone is adequately demonstrated, and the treatment of data, while generally sound, it could be further enhanced by the following considerations:
  4.1. In the calculation of the benefit-cost ratio of the initial investment, i.e. present value of losses avoided/total investment, the reviewer’s considered opinion is that the prices paid for purchase of the traps and the initial amount invested in research to develop the technology (said to be US$50,000) may not adequately define total costs. The authors correctly mention they excluded labor and indirect intellectual investments – which could be considerable. The cost of technology delivery apart from initial research costs is often quite considerable. The authors should make an attempt to estimate these, although understandably these are a complex mix of quantifiable and qualitative values that are not easy to calibrate accurately.
  4.2. In terms of costs avoided, it would also be instructive to attempt to compute all the previous direct and ancillary costs e.g. labor costs of the inspections that used to be performed before the traps became available, in addition to the directly attributed (avoided) costs of previous chemical control.
  4.3. Data on annual average yield is adequately treated. However, **price calculations **are based on the average monthly price published by the Center for Advanced Studies on Applied Economics (Cepea) (Cepea, 2015), corresponding to the average amounts in US$ paid to citrus growers per box, on credit. In this calculation, price stability is assumed. The authors need to mention if they took account or how they treated supply Vs demand pressure on price, or any subjectively negotiated discounts on price?
  The accuracy of monetary values is in part, but importantly, dependent on the accurate treatment of price data. On the pivotal question of price, the reviewer considered the following questions:
  - Is the average price decomposed into the different contributing variables?
  - What were the relative contributions of each monetary variable to the aggregated components?
  - What was the price elasticity?
  - to what extent did the assigned exchange rate affected the overall pricing?
  - how stable was the exchange rate over the entire reference period?
  4.4. In the overall analysis it would be good to capture the computation of price in an equation that shows the relative contribution of each monetary variable to the aggregated components since the monetary variables were derived from multiple independent (assumed non-synchronized) sources e.g. cepea), PTAX), etc.
  4.5 Secondly, in transforming nominal price data into real values using the General Price Index – Internal Availability (IGP-DI) the authors need to discuss the price elasticity and sensitivity to each contributing variable.
  4.6 Thirdly, still on the question of pricing, monetary variables were updated to values applicable in June 2014, and the final month’s PITAX exchange rate used. The authors need to demonstrate to what extent did the assigned exchange rate affected the overall pricing, and explain how stable the exchange rate had been for the entire reference period.
5. The authors have made an excellent attempt to compute the actual economic (transactional) value of the the *Gymnandrosoma aurantianum *sex pheromone. Globally, in the determination of the broader public goods the overall discussion of “how much is a pheromone worth?” could be further enriched if the authors also discussed in greater detail its:
  a) Functional value? In terms of environmental ecosystem services provided in addition to economic value?
  b) Evolutionary value of sex pheromones? Maintenance of ecological balance though managed species populations?

**Conclusion**: The paper is sufficient for indexation, with minor revisions addressing the concerns above.

We have read this submission. We believe that we have an appropriate level of expertise to confirm that it is of an acceptable scientific standard.

# Peer review: Referee response for version 1

**DOI:** 10.5256/f1000research.9897.r15137

**Published:** 2016-07-25

**Authors:** A. Cameron Oehlschlager

**Affiliations:** 1 ChemTica Internacional, Heredia, Costa Rica

This paper analyzes the monetary benefits accruing to citrus growers adopting a 1 trap / 10 ha pheromone trap monitoring program for citrus fruit borer *Gymnamdrosoma aurantianum* between 2001 and 2013.  The principle savings was in a  50% reduction in insecticide application accompanied by significantly lower fruit damage. This is the first time the economic analysis of long term pheromone trap monitoring to better time insecticide application has been conducted.  Because manpower costs of monitoring and spraying are not included, and because spraying uses much more manpower than monitoring, the actual additional income to citrus growers using the monitoring program is very likely more than estimated in this paper. The information in this paper is of significant interest to managers of agricultural enterprises.

I have read this submission. I believe that I have an appropriate level of expertise to confirm that it is of an acceptable scientific standard.
